# Core decompression with β-tri-calcium phosphate grafts in combination with platelet-rich plasma for the treatment of avascular necrosis of femoral head

**DOI:** 10.1186/s12891-022-06120-z

**Published:** 2023-01-18

**Authors:** Jinyang Lyu, Tiancong Ma, Xin Huang, Jingsheng Shi, Gangyong Huang, Feiyan Chen, Yibing Wei, Siqun Wang, Jun Xia, Guanglei Zhao, Jie Chen

**Affiliations:** 1 Department of Orthopaedic Surgery, Huashan Hospital Fudan University, 12Th Wulumuqi Middle Road, Jing’an District, Shanghai, China; 2 Fudan University, 220Th Handan Road, Yang’pu District, Shanghai, China; 3 Department of Orthopaedic Surgery, North Branch of Huashan Hospital Fudan University, 518Th Jingpohu Road, Bao’shan District, , Shanghai, China

**Keywords:** Core decompression, Platelet-rich plasma, Avascular necrosis of femoral head, Hip-preserving strategy, β-tri-calcium phosphate

## Abstract

**Background:**

This study was aimed to investigate whether the application of platelet-rich plasma (PRP) combined with β-tri-calcium phosphate (β-TCP) grafts after core decompression (CD) could improve the clinical outcomes of early stage of avascular necrosis of femoral head.

**Methods:**

Forty-five (54 hips) patients with Ficat-Arlet classification stage I-II treated by CD with β-TCP grafts with or without the application of PRP from July 2015 to October 2020 were reviewed. Group A (CD + β-TCP grafts) included 24 patients (29 hips), while group B (CD + β-TCP grafts + PRP) included 21 patients (25 hips). Visual analogue scale (VAS) score, Harris hip score (HHS), change in modified Kerboul angle and the hip joint survival were evaluated and compared between the groups. Patients had a mean follow-up period of 62.1 ± 17.2 months and 59.3 ± 14.8 months in group A and group B, respectively.

**Results:**

The mean VAS scores in group A was significantly higher than group B at the 6 months (2.9 ± 0.7 vs 1.9 ± 0.6, *p* < 0.01) and final follow up postoperative (2.8 ± 1.2 vs 2.2 ± 0.7, *p* = 0.04). The mean HHS in group A was significantly lower than group B at the 6 months (80.5 ± 13.8 vs 89.8 ± 12.8, *p* = 0.02). However, at the final follow up, there is no significant difference between the groups (77.0 ± 12.4 vs 83.1 ± 9.3, *p* = 0.07). The mean change in modified Kerboul angle was -7.4 ± 10.6 in group A and -19.9 ± 13.9 in group B which is statistically significant (*p* < 0.01). Survivorship from total hip arthroplasty were 86.2%/84% (*p* = 0.86) at the final follow up, which was not statistically significant. No serious complications were found in both groups.

**Conclusions:**

A single dose of PRP combined with CD and β-TCP grafts provided significant pain relief, better functional outcomes, and delayed progression in the short term compared to CD combined with β-TCP grafts. However, the prognosis of the femoral head did not improve significantly in the long term. In the future, designing new implants to achieve multiple PRP injections may improve the hip preservation rate.

## Introduction

Avascular necrosis of femoral head (ANFH) is recognized as a refractory disease which usually results in the dysfunction and deterioration of the hip joint [1]. An estimated 300,000 to 600,000 cases of ANFH are diagnosed in the United States, while the estimated cumulative number of patients diagnosed with ANFH in China has reached 8.12 million [2, 3]. High risk factors for the development of ANFH include corticosteroids application, alcohol abuse, trauma, smoking, hyperlipidemia and sickle cell disease [4]. Total hip arthroplasty (THA) is the golden standard treatment once the femoral head collapses [5]. However, ANFH occurs predominantly in patients aged from 20 to 50 years old, which means they could probably undergo at least one revision operation in the future due to the limited prothesis service life [6, 7].Consequently, a various of hip-preserving strategies such as core decompression (CD), vascularized cortical bone graft implantation, femoral osteotomies and so on, have been introduced to delay the progression of ANFH [8].

Historically, CD has been the most widely used hip-preserving surgical procedure since first described in 1960s [9]. It is believed that CD decreases the intramedullary pressure which plays an important role in the pathogenesis of ANFH. However, it is reported that CD alone had a failure rate of up to 77% in Ficat-Arlet Stage II after long-term evaluations [10]. This is because CD does not have superior capacity in facilitating the angiogenesis in the necrotic area. In an attempt to improve rates of hip preservation, platelet-rich plasma (PRP) has been proposed to treat the ANFH currently [11].

PRP, as a biological adjuvant, contains multiple high concentrations of growth factors, such as platelet-derived growth factor, transforming growth factor-β, basic fibroblast growth factor, endothelial growth factor, and vascular endothelial growth factor which can induce osteogenesis and angiogenesis by regulating the proliferation and differentiation of the bone marrow-derived mesenchymal stem cells and osteoblastic precursors [12–15]. In addition, PRP is safe to use as it is obtained autologously from the peripheral blood by centrifugation. Recently, PRP is being widely used in orthopaedics for its obvious advantages. Although increasing number of studies have reported excellent clinical outcomes combined with PRP in treating early stage ANFH patients [16, 17], the routine use was still restrained lacking the support of conclusive evidence. There is a need for further investigation on its short and long term outcomes in hip-preserving strategies.

In our clinical centre (Orthopaedics Department of Huashan Hospital Fudan University, Shanghai, China), the angio-conductive β-tri-calcium phosphate (β-TCP) grafts as described previously were chosen to fill in the tunnel left after CD [18]. To our knowledge, its use in combination with PRP after CD to treat ANFH has not been investigated in clinical study. One animal study using the ANFH model demonstrated that PRP combined with β-TCP, which can promote new bone formation and inhibit inflammatory response, showed higher efficiency in repairing ANFH than internal fixation alone [19].What would be the results from combining PRP with core decompression and β-TCP grafts in clinic? We presumed that the application of PRP could further (1) relieve the pain and improve the hip function of early stage ANFH; (2) retard the collapse of the femoral head.

## Materials and methods

### Patients

The study was performed according to the ethical standards of the Ethics Committee of the National Health Commission, and written approval was obtained from the Ethics Committee of Huashan Hospital Fudan University, Shanghai, China. All the patients provided written informed consent.

This study reviewed 52 consecutive patients undergoing CD + β-TCP grafts with or without the application of PRP from July 2015 to October 2020. All participants were classified by the modified Ficat-Arlet classification system [20] according to the plain radiographs of the hips at the anteroposterior and frog-leg lateral positions. This is the most widely used radiographic classification, where stage I presents normal or minor changes such as blurring of the trabecular pattern, stage II shows signs of a sclerotic rim or radiolucent subchondral crescent, while stage III and IV present the partial or complete collapse of the femoral heads respectively. Magnetic resonance with T1, T2 and STIR sequences were also examined and the modified Kerboul angle [21] was measured before operation.

The inclusion criteria were: (1) patients aged from 18–70 years old, of either sex; (2) ANFH diagnosis with Ficat-Arlet classification I-II stage(either unilateral or bilateral);(3) No history of hip trauma; (4) all the medical history and radiographic data available;(5) the minimum follow- up period > 2 years. The exclusive criteria were: (1) patients lost to follow up; (2) a history of hip surgery on the same side; (3) Ficat-Arlet classification III-IV stage;(4) patients with history of infections, tumours, rheumatoid arthritis, bone marrow suppression or patients in pregnancy. After screening, two patients were excluded because of previous hip surgery, two patients were excluded as the Ficat-Arlet classification reached III stage and three patients lost follow up. Finally, a total of forty-five (54 hips) patients were involved. Group A (CD + β-TCP grafts) included 24 patients (29 hips), while group B (CD + β-TCP grafts + PRP) included 21 patients (25 hips) (Table 1). Average age of the patients at the time of surgery was 45.8 years (range from 22 to 70 years, median 47 years) in group A and 39.6 years (range from 21 to 52 years, median 35 years) in group B. 19 patients (79.2%) in group A and 17 patients (81%) in group B had unilateral hip involvement, whereas bilateral involvement was seen 5 patients (20.8%) in group A and 4 patients (19.0%) in group B. In group A, 58.3% patients had the history of corticosteroids administration and 29.1% patients had the history of alcohol abuse. In group B, 66.7% patients had the history of corticosteroids application and 23.8% patients had the history of chronic alcoholism. We had 7 hips cases in stage I and 22 hips cases in stage II in group A, 6 hips cases in stage I and 19 hips cases in stage II in group B. The mean final follow-up periods were 62.1 ± 17.2 months and 59.3 ± 14.8 in group A and B respectively.

**Table 1:** Basic demographic and epidemiologic data

Variables	Group A (*N* = 24)	Group B (*N* = 21)
Gender (male/female)	16/8	16/5
Age at surgery (mean ± SD; years)	45.8 ± 11.7	39.6 ± 10.8
Hip involved		
Unilateral (%)	19(79.2%)	17(81.0%)
Bilateral (%)	5(20.8%)	4(19.0%)
Etiology		
Corticosteroids (%)	14(58.3%)	14(66.7%)
Alcohol (%)	7(29.1%)	5(23.8%)
Others (%)	3(12.6%)	2(9.5%)
Ficat-Arlet classification (hips)		
Class I (%)	7(24.1%)	6(24.0%)
Class II (%)	22(75.9%)	19(76.0%)
Follow-up (months)	62.1 ± 17.2	59.3 ± 14.8

*SD* Standard Deviation

### Platelet-rich plasma preparation

The platelet-rich plasma was prepared as per the instruction of the Platelet Rich Plasma Preparation Kits produced by Shandong Wego New Life Medical Devices CO., Ltd.After the induction of anesthesia, 30 ml peripheral venous blood was harvested using the Platelet Rich Plasma Preparation Kits The sample was centrifuged for 10 min at 1400 rpm to obtain the buffy coat, followed by second centrifugation for 5 min at 2350 rpm. After the supernatant was removed, the remaining was the PRP.

Calcium chloride and thrombin were added to keep the bio-activity of PRP.

### Surgical procedure

**Fig. 1:**
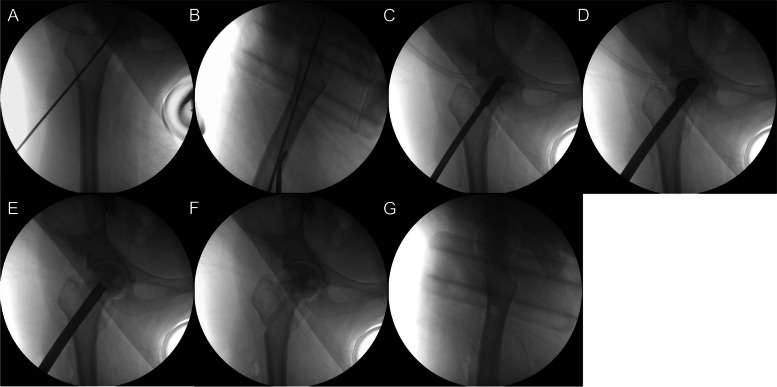
Intraoperative positioning by X-rays. The position of the guide wire was confirmed by anteroposterior (**A**) and lateral (**B**) X-rays. **C** A bone channel was reamed out over the K-wire to achieve core decompression. **D** Necrosis debridement was carried out with the reamer through progressive expansion of the blades. **E** The porous and dense granules previously mixed with bone sludge and pre-prepared PRP (Fig. 2) were grafted and impacted into the necrotic area. **F** and **G** The β-TCP rod was then inserted and implant positioning was confirmed by anteroposterior and lateral X-rays finally

**Fig. 2:**
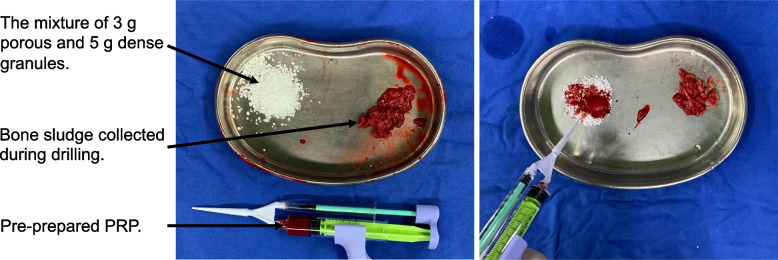
The porous and dense granules mixed with bone sludge, and then immediately mixed with pre-prepared PRP

The surgical procedure was performed as per the previous published study [18]. The procedure was performed on a traction table in the supine position under general anesthesia in all patients. A guide wire was inserted from the level of lesser trochanter, through the centre of the femur neck, to 5 mm below the cartilage of the femoral head (Fig. 1). The position of the guide wire was confirmed by anteroposterior and lateral X-rays. An approximately 1-2 cm skin incision was made along the femoral shaft, with the guide wire as centre; subcutaneous fascia and vastus lateralis were carefully cut layer by layer until reaching the bone. Then a bone channel of 8 mm diameter was drilled over the K-wire to have adequate core decompression. During drilling, bone sludge containing mesenchymal stem cells, stromal cells, and blood cells was collected, immediately mixed with 3 g porous and 5 g dense β-TCP granules [18] and in addition mixed with or without pre-prepared PRP (Fig. 2). Necrosis debridement was carried out with the reamer through progressive expansion of the blades. Using the graft delivery system, the above mixture was grafted and impacted into the necrotic area. The β-TCP rod (Shanghai Bio-Lu Biomaterials Co, Ltd) was then inserted and must extend at least to the two third of the femoral head to facilitate new tissue formation. After fluoroscopic confirmation of implant positioning, the incision was cleaned and sutured.

### Rehabilitation

All patients followed a standard post-operative rehabilitation program [18, 22]. Patients were kept non-weight-bearing on the operative side for three months and allowed to walk with the crutches. Partial weight-bearing was allowed for the subsequent four weeks and full weight-bearing was started on the fifth month. The exercise of the gluteal muscles and quadriceps was encouraged.

### Clinical assessment

Pain relief and function of the hip were assessed by visual analogue scale (VAS) score and Harris hip score (HHS) [23], respectively, at different time points(before surgery, at 6 months and > 2 years follow up). At each follow up, anteroposterior and frog-leg lateral radiographs were evaluated to monitor the collapse of the femoral head. The MRI images of the hips were followed up every year to assess the change of modified Kerboul angle compared with the pre-operative one.

### Statistical analysis

SPSS (version18.0; SPSS Inc, Chicago) was used to analyse the data. The Kaplan–Meier method was used to make survival curves in order to assess the survivorship free from THA. Comparison between the two groups was performed using independent samples t-test for continuous data and Mann–Whitney U test for non-parametric data. A *p* value less than 0.5 was considered significant.

## Results

The mean VAS score was 4.7 ± 0.9 in group A and 4.7 ± 0.8 in group B at the baseline (*p* = 0.98) and reduced to 2.9 ± 0.7 in group A and 1.9 ± 0.6 in group B at 6 months postoperative. At the final follow up, the VAS score has reduced to 2.8 ± 1.2 in group A and 2.2 ± 0.7 in group B. The mean VAS scores in group B was significantly lower than group A at 6 months (*p* < 0.01) and final follow up postoperative (*p* = 0.04) (Table 2).

**Table 2:** VAS score at the baseline and after intervention

Description(mean ± SD)	Group A	Group B	*p* Value
VAS Score preoperative	4.7 ± 0.9	4.7 ± 0.8	0.98
VAS Score at 6 months	2.9 ± 0.7	1.9 ± 0.6	< 0.01
VAS Score final	2.8 ± 1.2	2.2 ± 0.7	0.04

*VAS* Visual Analogue Scale, *SD* Standard Deviation

The mean HHS was 64.6 ± 4.1 in group A and 64.7 ± 4.7 in group B at the baseline (*p* = 0.92) and increased to 80.5 ± 13.8 in group A and 89.8 ± 12.8 in group B at 6 months postoperative. At the final follow up, the score reduced to 77.0 ± 12.4 in group A and 83.1 ± 9.3 in group B. The mean HHS in group B was significantly higher than group A at 6 months (*p* = 0.02). However, at the final follow up, there is no significant difference between the groups (*p* = 0.07) (Table 3).

**Table 3:** HHS Score At The Baseline And After Intervention

Description(mean ± SD)	Group A	Group B	*p* Value
HHS preoperative	64.6 ± 4.1	64.7 ± 4.7	0.92
HHS at 6 months	80.5 ± 13.8	89.8 ± 12.8	0.024
HHS final	77.0 + 12.4	83.1 + 9.3	0.07

*HHS* Harris Hip Score, *SD* Standard Deviation

The mean modified Kerboul angle was 202 ± 7.2° in group A and 203.9 ± 5.6° in group B at the baseline (*p* = 0.57) and reduced to 195.4 ± 9.8° in group A and 189.1 ± 16.0° in group B at final follow up postoperative. The mean change in modified Kerboul angle was -7.4 ± 10.6° in group A and -19.9 ± 13.9° in group B which is statistically significant (*p* < 0.01) (Table 4).

**Table 4:** Modified Kerboul angle at the baseline and after intervention

Description(mean ± SD)	Group A(°)	Group B(°)	*p* Value
Modified Kerboul Angle preoperative	202.8 ± 7.2	203.9 ± 5.6	0.57
Modified Kerboul Angle final	195.4 ± 9.8	189.1 ± 16.0	0.12
Change in the Modified Kerboul Angle	-7.4 ± 10.6	-19.9 ± 13.9	< 0.01

*SD* Standard Deviation

**Fig. 3:**
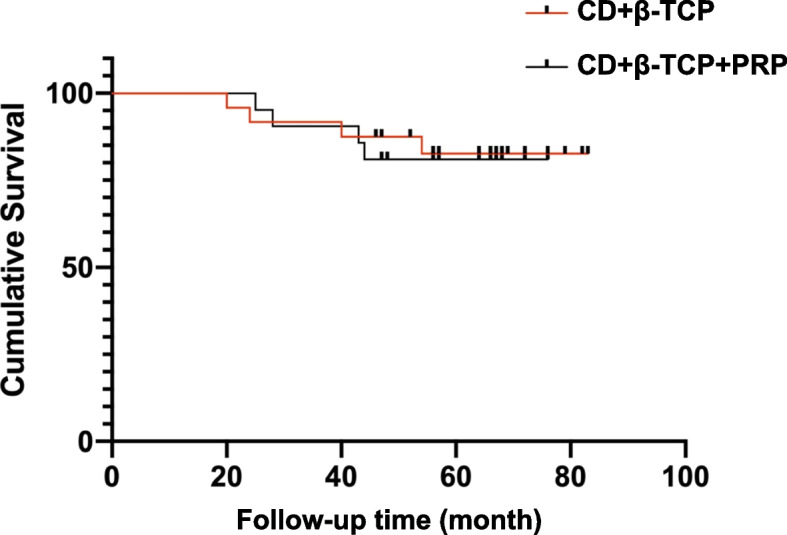
Cumulative survival of two groups during follow-up time

In both groups, THA occurred in four collapsed femoral heads at the final follow up. Survivorship from THA at the final follow were 86.2%vs.84% (*p* = 0.86); as shown by the Kaplan–Meier survival curve (Fig. 3), which was not statistically significant. All hips that had undergone THA were in Stage II of the Ficat-Arlet classification system. In group A, there were two failed hips caused by corticosteroids and two by alcohol, while in group B, there were three failed hips caused by corticosteroids and one by other reason. No serious complications were found in both groups.

## Discussion

In the present study, the clinical assessment data of 45 early stage ANFH patients who received CD plus β-TCP grafts with or without the application of PRP treatment from July 2015 to October 2020 was retrospectively analysed. Our study indicated that the application of PRP combining with CD and β-TCP grafts could significantly improve the symptoms and inhibit the progression of femoral head necrosis in the short term. Unfortunately, it did not significantly improve the rate of hip preservation.

The β-TCP material has been gradually studied and applied in ANFH because of their good biocompatibility, biodegradability, and osseointegration properties [24]. The β-TCP bio-ceramics grafts used in our study were designed and produced by Bio-Lu Biomaterial Co., Ltd. The pore size and interconnection diameter of the porous microstructure were optimized for a good compromise between angio-conductivity and mechanical property, which contribute significantly to the capacity of vascularization and osteogenesis. After implantation in the femoral head, the mechanical strength of the femoral neck and femoral head recovers gradually as the bio-ceramics degrade and new bone is formed, until the biomechanical support is fully restored.

Our team has achieved good results reporting an overall joint survival rate of 90.27% in the previous study of the application of bio-ceramic grafts prepared by β-TCP in the treatment of early femoral head necrosis [18].In our present study, the total hip survival rate was 86.2% at the mean time of 60 months follow-up. The overall survival rate were well-matched but the follow up time was much longer than the previous one. Wan et al. [22] compared the outcome of different bone grafts including the free fibular graft, the free vascularized fibular graft, autologous iliac bone, and β-TCP graft in early stage of ANFH. Satisfactory early-middle outcomes were shown in all the four bone graft implantation. However, as compared to other bone grafts, the β-TCP graft has the advantages of shorter operation time and lesser blood loss. In addition, the application of β-TCP grafts would not cause any damage to other parts of the body.

.PRP is defined as the plasma fraction of autologous blood with platelet concentrations above baseline. The normal platelet concentration is 200,000–450,000/ml. Studies have shown that clinical efficacy can be expected to increase by at least 4 times baseline (1 million/ml). Platelets play an important role in the normal healing response through the local secretion of growth factors and the recruitment of repair cells [25]. PRP may exert the beneficial effect in ANFH in the following ways: inducing angiogenesis and osteogenesis to accelerate bone healing, inhibiting inflammatory reactions in necrotic lesions, and preventing apoptosis induced by glucocorticoids [26, 27].

Theoretically, the CD combined with the β-TCP grafts provided the ‘land’ for the bone regeneration. For better healing, extra ‘fertilizer’–-PRP, is needed. Our study shows that the application of combining PRP with CD and β-TCP grafts can significantly improve the symptoms including VAS scores at 6 months (*p* < 0.01), HHS (*p* = 0.02) and inhibit the progression of femoral head necrosis in the short term with change in the modified Kerboul angle (p < 0.01) as compared with group A. Unfortunately, it does not significantly improve the rate of hip preservation at the finial follow up (*p* = 0.86).

Aggarwal et al. [25] conducted a prospective randomized double blinded comparative study in 40 early stage ANFH patients to investigate the efficacy of CD + PRP compared with CD alone, and the results showed that PRP use after CD provides significant pain relief, better midterm functional outcome, retards the progression, and enhances the survivorship free from reoperation for hip arthroplasty and femoral head collapse than CD alone. The survivorship from femoral head collapse in PRP + CD group/ CD group was 84%/68%. Compared with the CD + β-TCP group in our study, the survival rate in CD group was much lower, which indicates the significance of the mechanical support of β-TCP grafts and the adjuvant function of PRP.

In another study, Marco Grassi et al. [16] reported that core decompression combined with PRP could be indicated as a treatment for the I and II stages of ANFH in a prospective not controlled study The overall survival rate was 80% at two years follow up and 47% at five years follow up. Our hip-preserving strategy with the combination of CD + PRP + β-TCP presented better survival rate both at the two years and five years follow up.

In a prospective comparative study [28], forty-four patients (57 hips) with early ANFH were randomized into two groups: group A received CD, autologous bone marrow buffy coat (BBC) and β-TCP grafts; group B received treatment of CD with β-TCP grafts. BBC is obtained from bone marrow and it has stem cell activity to promote cell proliferation. The survival rate in group A was 95.5%, which is much higher than our results in PRP group. However, in this study, the patients age was much younger and the follow up time was relatively shorter than ours. Similar survival rate was reported in group B at the final follow up as compared to our study in CD + β-TCP group. Compared with BBC, the preparation of PRP is more minimally invasive and convenient.

Our result suggests that single-dose PRP effectively inhibits the progression of femoral head necrosis (modified Kerboul angle, *p* < 0.01), but also suggests that single-dose PRP may not be able to improve hip preservation rate, even with early stage ANFH. In the latest case report [29], the authors presented a case of ARCO IV glucocorticoid-induced ANFH received five consecutive ultrasound-guided intra-articular injections of PRP. Surprisingly, at 9-month follow-up, clinical and radiological reassessments demonstrated favorable outcomes. This case highlights the therapeutic potential of multiple times PRP injections for ANFH, even late-stage ANFH. At present, there is no relevant literature to propose a new solution. On the basis of the previous research, our team designed a femoral head implantation catheter technology (an invention patent has been applied for), hoping to achieve continuous input of PRP after CD to obtain better therapeutic effect and inhibit the progress of femoral head necrosis, and improve the hip preservation rate.

The results of the current series should be interpreted acknowledging certain limitations. Our study was retrospective without rigorous paired controls, and the strength of the evidence is limited. Second, the small sample size of this study still requires a large number of patients to support the further advantages of CD and β-TCP grafts combined with PRP. The specific location and size of the lesion were not analysed. In addition, some patients had bilateral disease; however, we analysed each hip independently. Study results may have been different if survival without THA, reoperation, or femoral head collapse was analysed for only one hip per patient.

## Conclusions

A single dose of PRP combined with CD and β-TCP grafts provided significant pain relief, better functional outcomes, and delayed progression in the short term compared to CD combined with β-TCP grafts. However, the prognosis of the femoral head did not improve significantly in the long term. In the future, designing new implants to achieve multiple PRP injections may improve the hip preservation rate.

## Data Availability

The datasets used and/or analyzed during the current study available from the corresponding author on reasonable request.

## Abbreviations

PRP: Platelet-rich plasma
β-TCP: β-Tri-calcium phosphate
CD: Core decompression
VAS: Visual analogue scale
HHS: Harris hip score
ANFH: Avascular necrosis of femoral head
THA: Total hip arthroplasty
BBC: Bone marrow buffy coat
